# Pharmacotherapeutic Potential of *Aloe secundiflora* against Colorectal Cancer Growth and Proliferation

**DOI:** 10.3390/pharmaceutics15051558

**Published:** 2023-05-22

**Authors:** John M. Macharia, Veronica Ngure, Barnabás Emődy, Bence Király, Zsolt Káposztás, Nóra Rozmann, Attila Erdélyi, Bence Raposa

**Affiliations:** 1 Doctoral School of Health Sciences, Faculty of Health Sciences, University of Pẻcs, 7624 Pecs, Hungary; johnmacharia@rocketmail.com (J.M.M.);; 2 School of Science and Applied Technology, Laikipia University, Nyahururu P.O. Box 1100-20300, Kenya; 3 Faculty of Health Sciences, University of Pẻcs, 7624 Pecs, Hungary

**Keywords:** *Aloe secundiflora*, colorectal cancer, phytotherapy, plant, biomolecules

## Abstract

*Aloe* species are widespread and diverse in African ecosystems, and this commonly correlates to their habitual use as reservoirs of herbal medicine. The side effects associated with chemotherapy and the development of antimicrobial resistance to empirically used antimicrobial drugs are substantial, paving the way for novel phytotherapeutic approaches. This comprehensive study aimed to evaluate and present *Aloe secundiflora (A. secundiflora)* as a compelling alternative with potential benefits in colorectal cancer (CRC) treatment. Important databases were systematically searched for relevant literature, and out of a large collection of 6421 titles and abstracts, only 68 full-text articles met the inclusion criteria. *A. secundiflora* possesses an abundant presence of bioactive phytoconstituents in the leaves and roots, including anthraquinones, naphthoquinones, phenols, alkaloids, saponins, tannins, and flavonoids, among others. These metabolites have proven diverse efficacy in inhibiting cancer growth. The presence of innumerable biomolecules in *A. secundiflora* signifies the beneficial effects of incorporating the plant as a potential anti-CRC agent. Nonetheless, we recommend further research to determine the optimal concentrations necessary to elicit beneficial effects in the management of CRC. Furthermore, they should be investigated as potential raw ingredients for making conventional medications.

## 1. Introduction

### 1.1. The Biodiversity of Aloe Plant Species

*Aloe* species are abundant and diverse in African landscapes, and this frequently translates to their regular use as a source of phytotherapeutic medicine to support human health and wellbeing. They have also occasionally been mentioned as having utility in ethnoveterinary medicine by African ethnic groups [1]. *Aloe* L. (Asphodeloideae) is a blooming succulent genus with about 500 species, including trees, shrubs, and perennials. The genus is primarily native to Africa, with species also found in the Arabian Peninsula and Jordan, as well as on various islands off the African coast, including Madagascar [2].

While species overlap exists throughout regions, Southern Africa (Angola, Botswana, Lesotho, Malawi, Mozambique, Namibia South Africa, Swaziland, Zambia, and Zimbabwe) has the greatest biodiversity of *Aloe* species, with roughly 290 species reported [3]. East Africa (Burundi, Djibouti, Eritrea, Ethiopia, Kenya, Rwanda, Somalia, South Sudan, Tanzania, and Uganda) has a remarkable *Aloe* biodiversity, with around 200 species, while Madagascar and the Indian Ocean Islands have about 90. The Arabian Peninsula is home to roughly 50 species, and central and western African regions are home to nearly 30 species [2,3]. The perennial, drought-resistant, and succulent nature of the plant genus *Aloe* allows it to colonize dry areas. It can endure prolonged desiccation because its thick leaves can store a significant amount of water [4].

### 1.2. Ethnomedicinal Utility of the Plant Genus Aloe

Since 1500 BCE, aloe species have been crucial to history’s medical and economic development, and the gel found inside their leaves has been utilized to treat both human and animal ailments [5]. Herbalists from a wide range of African cultural groups have used plants in the genus *Aloe* for a wide range of medicinal applications. Since ancient times, traditional medicine has made extensive use of the species *Aloe vera* (L.) Burm.f. (Asphodelaceae) [6]. Its mucilaginous fluid is administered to wounds and cuts in rural areas of the continent to suppress infections and promote healing [7]. *Aloe* spp. have been reported to exhibit anti-inflammatory, immunostimulatory, and cellular growth-stimulating properties [8]. Furthermore, their extracts have been reported to have antibacterial, antiviral, antifungal, antiprotozoal, and purgative effects, along with cell proliferation, anticancer properties, and toxicological activities for utility in pharmacology [8,9,10,11]. The methanol extract of *A. vera* gel contains an assortment of active ingredients with antimicrobial activities that can be employed as antimicrobial agents in new therapeutic preparations for the treatment of infectious illnesses in humans [9].

One of the most frequently employed techniques in both traditional and modern medicine is skin healing and tissue regeneration. In vitro research, followed by experimental and clinical trials, has verified empirical data [11]. According to Liang et al., adding gel to wound dressings would be an easy and consistent technique for the use of *A. vera.* Although inflammation is a normal part of healing, *Aloe’s* anti-inflammatory properties appear to promote tissue recovery [12]. Although *Aloe* leaf gel has been used for many years, little is known about the pharmacological effects of its constituent parts [6]. It has been stated that the leaf gel of many *Aloe* species exhibits anti-inflammatory qualities, and research suggests that this property may be specifically related to TLR4-mediated reactions. It has also been shown that *Aloe* species at a gel concentration of 0.2% significantly reduces the NF-κB activity with LPS but has no discernible effect on FSL-1. Moreover, it appears that *Aloe* gel extracts are more effective at inhibiting LPS’s ability to activate the TLR4 pathway [6]. A recent assessment on *Aloe* and skin protection activities found that the constituents have not been sufficiently investigated to draw definite conclusions about their effects [13], and this warrants more investigative research studies for clarity, understanding, and validation.

### 1.3. The Biodiversity and Botanical Description of Aloe secundiflora

**Figure 1:**
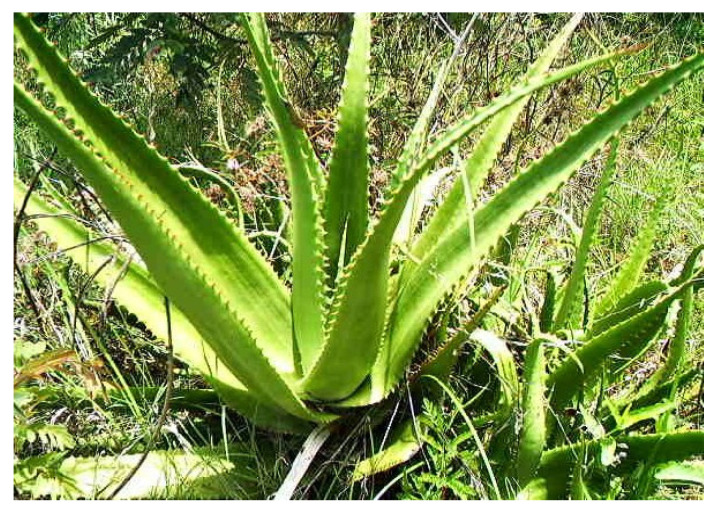
The occurrence of *Aloe secundiflora* naturally growing in the wild without human interference in Kenya [15].

The Asphodelaceae family’s largest genus, *Aloe*, contains more than 400 species that range in size from small shrubs to large trees and are found throughout dry regions of Africa, India, and other regions [7]. The diversity of *Aloe* is greatest in South Africa. *Aloe secundiflora* (Asphodelaceae) shrubs are common in open grasslands and bushlands in countries in Africa such as Ethiopia, Sudan, Kenya, and Tanzania. Typically, they have an acaulescent rosette of glaucous green leaves that expand and have a glossy sheen. The tips of the leaves are usually somewhat recurved (Figure 1). On their leaves—especially the undersides—young plants frequently have dots. Up to 20 spreading branches, each with a cylinder raceme of pink-red flowers, can be seen on the tall (1 m) erect inflorescence [3,7,14].

### 1.4. The Epidemiology and Risk Factors for Colorectal Cancer (CRC)

Cancer is the second leading cause of death, after cardiovascular illnesses. In Western societies, colorectal cancer is currently the third leading cause of cancer-related fatalities [16]. In underdeveloped nations, cancer deaths account for over 70% of fatalities [17]. Even though many CRCs are sporadic, a substantial percentage (5–6%) have a distinct hereditary relationship. CRC is the most common type of gastrointestinal (GI) tract malignancy [18]. Unhealthy diets deficient in whole grains, vegetables, and fruits, along with high-fat diets, gender, race, age, family history, and location, are factors contributing to the high incidence of colon cancers. Behavioral risks such as lack of exercise, smoking, and excessive alcohol consumption have also been reported as significant contributing factors [19]. Macharia et al. have also indicated exposure to pathogenic organisms and heavy metals as notable risk factors for CRC [18].

### 1.5. Current Therapeutic Applications and Future Approaches for Sustained CRC Management

Deciphering tumorigenic pathways will lead to a better understanding of the metabolic regulatory mechanisms used by quickly proliferating malignant cells, along with the development of more potent cancer therapeutics [20]. Despite the complexity and lack of clarity around the pathophysiology of colorectal cancer (CRC), interactions between risk factors seem to be crucial to the emergence and development of the disease.

The tumor microenvironment (TME) is made up of the extracellular matrix, the stromal and immune cells that it surrounds, and metabolites and signaling molecules in the intercellular space [21]. For cell proliferation, migration, invasion, and metastasis to increase from the primary tumor location, a TME must be established [22]. Over the past 10 years, the use of lactic acid bacteria (LAB) prebiotics and probiotics to alter the tumor microenvironment (TME) has been increasingly popular [23,24]. Cytotoxic medicines, immunotherapy, chemotherapy, resection, targeted therapy, and radiotherapy are some of the standard therapeutic management and therapies for CRC [18]. Antiangiogenic drugs may also be utilized to treat and stop the spread of malignant cells in addition to these treatments. The advent of several CRC medicines has failed to decrease the mortality of people suffering from CRC, due to the high prevalence of metastasis. Techniques used to prevent CRC are often based on approaches for detecting adenomatous polyps that are pre-CRC abrasions for colon cancer [25].

Currently, a variety of chemotherapeutic medications and diets are used to treat CRC, in addition to the patient’s unique characteristics. First-line chemotherapy is administered using the drugs folfiri (levoleucovirin, 5-FU, and irinotecan) and folfox (oxaliplatin, fluorouracil, and formyltetrahydrofolate) in conjunction with cetuximab, panitumumab, or bevacizumab [26]. Panitumumab and bevacizumab have been found to increase the effectiveness and safety of CRC treatment [25].

Mucositis, nausea, vomiting, and gastrointestinal toxicity are the most prevalent side effects of folfiri treatment. Irox is preferred when patients are intolerant to 5-FU, demonstrating that it is an effective second-line treatment choice for these patients with advanced CRC [26]. Phytochemicals and their derivatives are playing a larger role in the treatment and prevention of CRC because of the extreme side effects of these chemotherapeutic medications [27]. The side effects linked with chemotherapy are substantial and occur after healthy normal cells experience damage [28], paving the way for novel phytotherapeutic approaches as potent and safe alternatives. Plant-based natural products have been a prolific source of new anti-colon-cancer drugs, accounting for approximately 50% of all commonly used chemotherapeutic treatments, either directly or indirectly. Significantly, several of the pharmaceuticals that are currently in the market are produced from plant sources, which are employed as a good supply of drugs [29]. Plants and products derived from plants continue to be crucial components of the medical and healthcare systems in developing nations. In non-industrialized communities, the use of medicinal plants (herbs) to treat ailments is practically universal and is frequently less expensive than using costly conventional medications [30]. Aspirin, antimalaria drugs, anticancer drugs, and *digitalis* are examples of current medications that have been developed from plant origins [29].

It is against this backdrop that this updated review aims to evaluate data and present *Aloe secundiflora* (*A. secundiflora*) as a strong alternative with potent beneficial effects in colorectal cancer (CRC) treatment. However, as established, scarce documentation is available on this important plant species, while to the best of our knowledge no in vivo or in vitro CRC experimental studies have been documented explicitly. This is therefore the first updated review reporting on the active potential of *A. secundiflora* in CRC management in humans.

## 2. Methods

### 2.1. Applied Study Design and Electronic Databases

**Figure 2:**
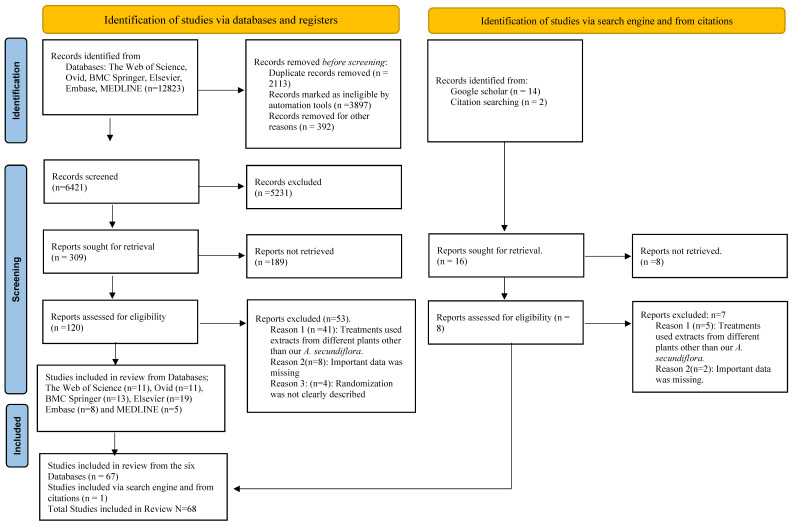
PRISMA flow diagram with slight modifications for systematic reviews conducted on six established databases and a search engine.

In this study, data on the botanical descriptions, ethnomedical applications, phytoconstituents, and pharmacological activities of *Aloe secundiflora* plant species against CRC growth and proliferation were compiled using a comprehensive review methodology. The Web of Science, Ovid, BMC, Springer, Elsevier, Embase, and MEDLINE databases were systematically searched for relevant literature to identify primary studies evaluating the effects of *A. secundiflora* on CRC carcinogenesis, in adherence with the PRISMA guidelines [31], but with slight modifications (Figure 2). Google Scholar was helpful in augmenting additional knowledge. The authenticity of Google Scholar articles was established by correlating them with the relevant publications considering their true publisher [32,33].

### 2.2. Review Question and the Screening Criterion

A review question was developed to make it simpler to use the search criteria. “Are Aloe secundiflora active metabolites effective against colorectal cancer growth?” was the explicit question. The authors independently assessed only English research titles and abstracts from primary studies after developing the review question for possible inclusion in the study [34,35]. All studies pertaining to conditions other than colorectal cancer and studies focusing on the small intestinal mucosa were expressly excluded from the analysis. Titles and abstracts that satisfied the minimal satisfactory standards were chosen for full-text article examination and subsequently used in the present review after being deemed appropriate by five authors. The authors’ independence was necessary when selecting whether to use the enrolled articles to minimize the chance of bias. [34,35]. Out of a large collection of 6421 titles and abstracts, only 68 full-text articles met the inclusion criteria (Figure 2).

### 2.3. Effective Search Strategy

A systematic search criterion was created to ensure that the screening method was effective. The MeSH terms “development”, “growth”, “proliferation”, “anti-cancer”, “bioactivity”, “physiological activity”, “phytochemicals”, “pharmacological activities”, and “multiplication” were combined with the keywords “colon cancer”, “colorectal”, “adenocarcinomas”, “polyps”, “colon tumor”, and “colorectal tumor”, along with their synonyms. These terms were finally combined with the plant species under review, “*Aloe secundiflora*”. A systematic search of the relevant literature was performed considering all articles published between the years 2005 and 2022. However, greater emphasis was placed on articles published within the last 10 years to utilize recently published articles.

## 3. Bioactive Compounds/Substances Present in *Aloe secundiflora*

**Figure 3:**
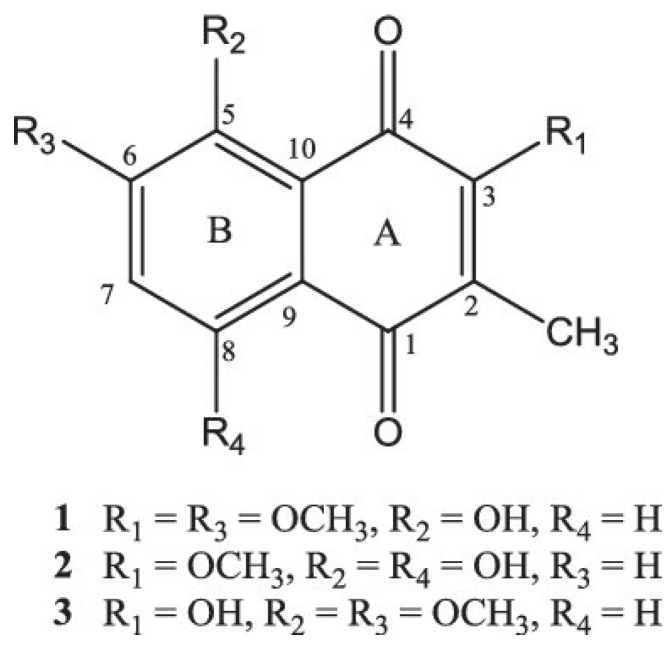
The chemical structure of naphthoquinones common in the roots of *A. secundiflora* [37].

**Figure 4:**
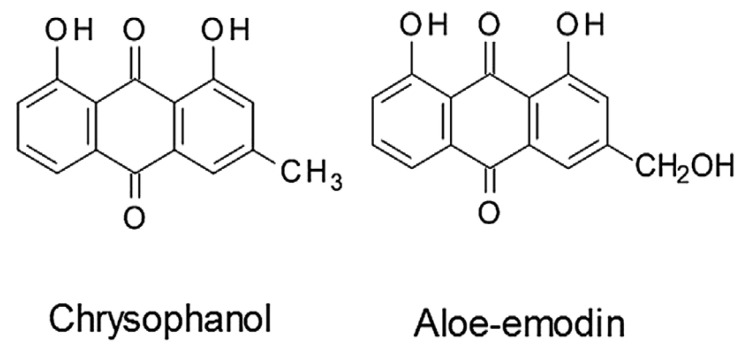
The selected chemical structures of anthraquinones, chrysophanol, and aloe-emodin common in the roots of *A. secundiflora* [38].

*Aloe* species have been used in ethnopharmacology, and studies on their phytochemical and pharmacological properties have produced several active ingredients. Herbalists have historically employed them to manage a variety of illnesses [7]. *Aloe* species are abundant natural sources of bioactive substances, with anthraquinones making up most of them [11]. Preliminary phytochemical analysis of *A. secundiflora* has indicated the presence of terpenes, flavonoids, and tannins (Table 1) in leaves [36], and naphthoquinones in roots (Figure 3), [37]. Anthracenedione (9,10-anthracenedione) is the core of anthraquinones (Figure 4), which are structurally related to anthracene [38]. Sometimes, they are referred to as 9,10-dioxoanthracene. Often, anthraquinones are found in their glycosidic forms. These components give plants their pigments and are frequently used as natural dyes [38]. Documented evidence demonstrates that vitamins A, B, B2, B6, B12, and E are present in *Aloe* species, making them a potent anti-inflammatory and antioxidant agent [3]. Enzymatic amylase, lipase, and carboxypeptidase present in the plant’s extracts help in the synthesis of fatty acids, sugars, and starch [10]. Gels have been reported to contain mineral ions, such as Mg^2+^, Zn^2+^, Ca^2+^, K^+^, Na^+^, Fe^2+^, P^−^, Mn^2+^, Cu^2+^, and Mo^4+^ [11].

## 4. Mechanisms of Carcinogenesis and the Potential Inhibitory Role of *A. secundiflora* in CRC Growth and Proliferation

**Figure 5:**
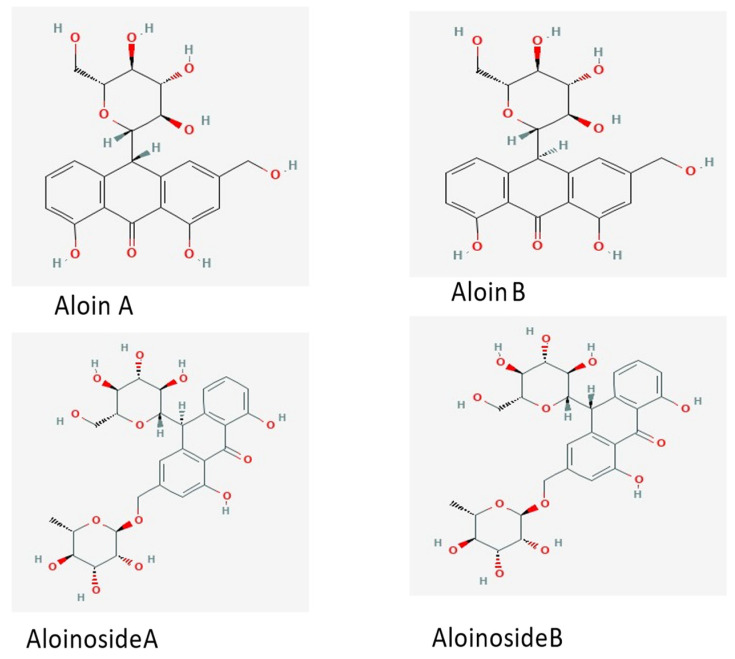
The chemical structures of aloin derivatives (anthrones) commonly found in the leaves of *Aloe* [42,43].

*Aloe* sp. have been found to be beneficial in treating a variety of malignancies that affect multiple organs, including the colon, according to a review by Singab et al. [41]. For the treatment of gastrointestinal malignancies, *Aloe* is seen as a possible medication [11]. Aloe-inhibitory emodin’s effect on the proliferation and migration of gastric tumor cell lines is dose-dependent. Aloin (C_21_H_22_O_9_)—a yellow chemical—is a combination of the diastereoisomers aloin A and aloin B. Aloin (AL)—an anthrone C-glucoside with a molecular weight of 418—is the primary phytoconstituent of *Aloe* (Figure 5). Aloin is used in pharmacology for many different purposes, including as a laxative [29].

AL has been reported to have effective activity in reducing tumor angiogenesis and growth, by preventing STAT3 activation in CRC cells in in vivo and in vitro experimental treatments [44]. In the experiment, the authors reported that Western blotting revealed that AL prevented endothelial cells from phosphorylating STAT3 and activating the EGF receptor (VEGFR2). Moreover, the activation of STAT3-regulated anti-apoptotic (Bcl-xL), cell proliferation (c-Myc), and angiogenic (VEGF) proteins, as well as the constitutively activated STAT3 protein, were all downregulated in response to AL in human SW620 cancer cells. In line with these findings, AL significantly decreased the tumor size and weight in mouse xenografts while inhibiting tumor cell viability and inducing cell apoptosis in vitro. This was done without causing any obvious harm [44]. The human gastric cancer MKN45 cell line showed anticancer activity for aloe-emodin and emodin [45].

Chrysophanol, sometimes referred to as 1,8-dihydroxy-3-methyl-anthraquinone and chrysophanic acid, is a naturally occurring anthraquinone in *Aloe* plants [46]. Its toxicity to aggressive human cancer cell lines (HepG2, HCT-8, A549, SGC7901, and MDAMB-231) was studied by Yao et al. [47], who demonstrated that there was no detectable cytotoxicity to the cells. Chrysophanol did, however, exhibit a time-dependent suppression of HepG2 cell viability, with the highest inhibition at 10 μm and a small waning of the potency of the inhibition at concentrations between 20 and 60 μm. Exposure of HL-7702 cells to chrysophanol, however, did not appear to cause any cytotoxicity at concentrations between 0 and 100 μm. In another study, Pandith et al. [48] discovered that chrysophanol at doses of 10, 50, and 100 g/mL inhibited the proliferation of cancer cells. Chrysophanol had little impact on colon cancer and breast cancer cell lines (MCF-7, T47D), according to other authors (HCT-116) [49]. The treatment’s dosage could unquestionably determine the recorded minimal effect. Chrysophanol caused considerable increases in the enzymes extracellular signal-regulated kinase (ERK1/2), p90 ribosomal protein S6 kinase (P90RSK), and protein kinase B (AKT), as well as ROS creation and mitochondrial malfunction [46].

The proangiogenic factor vascular endothelial growth factor (VEGF) is important for the development of tumor vascularity [50]. The main VEGF receptor and the key player in VEGF-induced angiogenesis pathways is vascular endothelial growth factor receptor 2 (VEGFR2) [51]. When dormant endothelial cells are stimulated, VEGFR2 signaling causes several downstream mediators to become active, which then promotes cell proliferation, migration, invasion, and differentiation into capillary-like structures [52]. Recent research has shown that VEGFR2-mediated signaling—particularly signal transducers—is frequently linked to a worse prognosis and is highly implicated in a wide range of human malignancies and STAT3, which is a transcription activator [53,54].

One of the latent self-signaling transcription factors in the cytoplasm that is triggered by specific cytokines (such as IL-6) and progenitor cells is STAT3 (e.g., VEGF). The transcription of responsive genes encoding apoptotic cell death inhibitors (e.g., Bcl-xL, Bcl-2) and inducers of angiogenesis (e.g., VEGF) is modulated by the activation of STAT3 homodimerization and nuclear translocation [55]. These genes are involved in cell proliferation, survival, differentiation, programmed cell death, metastatic spread, angiogenesis, human defense evasion, and drug resistance [56]. More recently, the scientific literature has been filled with evidence showing that inhibiting constitutive STAT3 signaling effectively prevents tumor growth and induces apoptosis [55,57]. Direct targeting of the STAT3 signaling cascade has been an appealing therapeutic target for pharmacological intervention due to the carcinogenic function of STAT3 and the promise of its inhibition [44]. Recently, plant-based substances with minimal adverse effects have been found to block angiogenesis and target STAT3 [57].

Even at high amounts, AL may be one effective, inexpensive, and safe oral medication used in clinical practice for the treatment and prevention of cancer [44]. Future research will clarify the therapeutic potential of AL in treating human colorectal cancer through both experimental and clinical trials, such as those examining its interaction with conventional chemotherapeutics. In addition, research published in vitro and in vivo has demonstrated that chrysophanol can control the expression of several genes and proteins, including GRP78, p-eIF2a, CHOP, caspase-12, Drp1, PTP1B, PAI-1, Bcl-2/Bax, and caspase-3. Chrysophanol also influences the signaling pathways for NF-jB, MAPK, PI3K/AKT, and PPARc [46].

**Figure 6:**
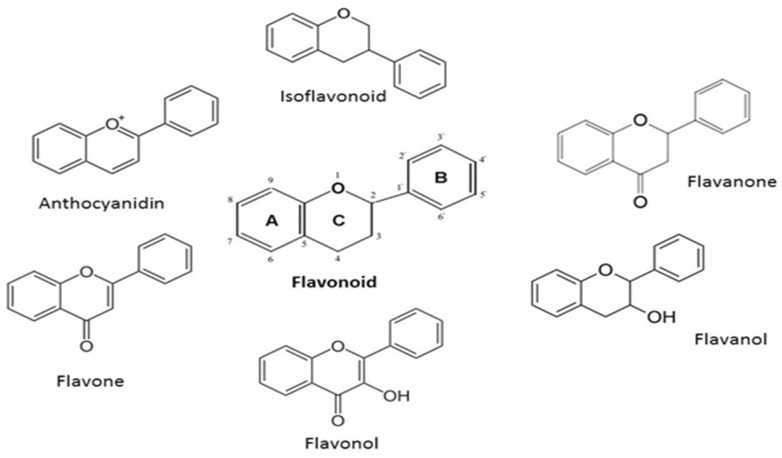
The different derivatives of flavonoids commonly found in *Aloe* and that have been reported to elicit significant pharmacotherapeutic effect [58].

The primary flavan skeleton, which is a 15-carbon phenylpropanoid chain (C6-C3-C6 system) that comprises two aromatic rings (A and B) connected by a heterocyclic pyran ring (C), is shared by all flavonoids (Figure 6) [58].

Flavonoids have been found to have a wide range of anticancer actions, including modulating the activity of enzymes that scavenge reactive oxygen species (ROS), participating in cell-cycle arrest, inducing apoptosis and autophagy, and reducing the proliferation and invasiveness of cancer cells [58]. In terms of maintaining the balance of reactive oxygen species (ROS), flavonoids operate as antioxidants under normal circumstances and as powerful pro-oxidants in cancer cells, where they activate apoptotic pathways and suppress pro-inflammatory signaling pathways. There is growing evidence that several flavonoids have anticancer properties, although the underlying molecular pathways are still not fully understood [58,59,60,61,62].

Due to the presence of phenolic hydroxyl groups, flavonoids have the capacity to stabilize free radicals and can directly scavenge ROS and chelate metal ions [63]. The activation of antioxidant enzymes, inhibition of pro-oxidant enzymes, and stimulation of the synthesis of antioxidant enzymes and phase II detoxification enzymes are all examples of indirect flavonoid antioxidant actions [63,64]. Flavonoids’ anticancer actions entail both antioxidant and pro-oxidant activity [64]. Owing to their antioxidant qualities, flavanols have been shown to protect against colon cancer [65]. Quercetin—a flavonoid—has strong chemopreventive effects on cancer [66,67]. By generating ROS, the flavonol kaempferol has been reported to activate caspases and induce apoptosis in colorectal cancer [68].

It is crucial to understand that *A. secundiflora* has similar pharmacotherapeutic characteristics to other species of the same genus, *Aloe.* They can thus be utilized for potential intervention and management of CRC in predetermined concentrations based on the extensive documented evidence on the pharmacological activity of the plant genus *Aloe*. We advise additional research to establish the proper concentrations and levels required to evoke positive effects in CRC management in humans, owing to the lack of proven amounts and specified concentration measurements.

## 5. Conclusions

The wellbeing of mankind is significantly impacted by the molecules in natural products, which have an evolutionary structure. Numerous secondary metabolites are synthesized by the biosynthetic machinery of nature, and each of them exhibits unique biological characteristics that make it desirable to use them as pharmaceutical structural models or as health products. It is crucial to understand that *A. secundiflora* contains similar pharmacotherapeutic characteristics to other identified species of the genus *Aloe.* It can thus be utilized for potential intervention and management of CRC in predetermined concentrations based on the extensive documented evidence on the pharmacological activity of the plant genus. The presence of innumerable biomolecules in *A. secundiflora* signifies the usefulness of incorporating this plant as a potential anti-CRC agent with certain phytotherapeutic effects. Despite the positive effects recorded in ethnoveterinary applications of *A. secundiflora*—and particularly in chickens—this is the first unique review detailing its potential application in human health, with specific emphasis on colorectal cancer. This is of course due to the abundant presence of bioactive phytoconstituents common in the leaves and roots of the perennial, evergreen, and succulent plants.

Nonetheless, before recommending *A. secundiflora* for clinical use, due to the lack of established quantities and concentration measurements, we recommend further research to determine the minimal or optimal concentrations/levels necessary to elicit beneficial effects in CRC management in humans, whether individually or in combination. Additionally, this important plant may be investigated as a potential source of raw ingredients for making conventional medications.

## Figures and Tables

**Table 1:** Bioactive compounds present in *A. secundiflora*.

Plant Part/Organ	Bioactive Compounds	References
**Roots**	Anthraquinones (chrysophanol, helminthosporin, aloe-emodin, aloesaponarin II and aloesaponarin I), laccaic acid D, methyl ester, and asphodelin. Naphthoquinones (5-Hydroxy-3,6-dimethoxy-2-methylnaphthalene-1,4-dione and 5,8-dihydroxy-3-methoxy-2-methylnaphthalene-1,4-dione)	[4,36,37,38]
**Leaves**	Phenols such as anthrones (aloenin, aloenin B, isobarbaloin, barbaloin, and other aloin derivatives), chromones, and phenylpyrones, Alkaloids, saponins, tannins, flavonoids (nthoxanthins, flavanones, flavanols, flavans, and anthocyanidin), steroids, cardiac glycosides, aloeresin, anthraquinones, aloin, hydroxyaloins, polyphenols, terpenoids	[4,36,38,39,40]

## Data Availability

No new data were created or analyzed in this study. Data sharing is not applicable to this article.
